# The regulation of circadian entrainment in mice by the adenosine the *A*_*2A*_ */A*_*1*_ receptor antagonist CT1500

**DOI:** 10.3389/fphys.2022.1085217

**Published:** 2022-12-20

**Authors:** Aarti Jagannath, Simona Di Pretoro, Farid Ebrahimjee, Suzanne Ftouni, Lewis Taylor, Russell G. Foster, Sridhar Vasudevan

**Affiliations:** ^1^ Sir Jules Thorne Sleep and Circadian Neuroscience Institute (SCNi) and Kavli Institute for Nanoscience Discovery, Dorothy Crowfoot Hodgkin Building, Nuffield Department of Clinical Neurosciences, University of Oxford, Oxford, United Kingdom; ^2^ Department of Pharmacology, University of Oxford, Oxford, United Kingdom

**Keywords:** circadian rhythms, pharmacology, light, entrainment, sleep

## Abstract

Circadian entrainment in mice relies primarily on photic cues that trigger the transcription of the core clock genes *Period1/2* in the suprachiasmatic nucleus (SCN), thus aligning the phase of the clock with the dawn/dusk cycle. It has been shown previously that this pathway is directly regulated by adenosine signalling and that adenosine A_2A_/A_1_ receptor antagonists can both enhance photic entrainment and phase shift circadian rhythms of wheel-running behaviour in mice. In this study, we tested the ability of CT1500, a clinically safe adenosine A_2A_/A_1_ receptor antagonist to effect circadian entrainment. We show that CT1500 lengthens circadian period in SCN *ex vivo* preparations. Furthermore, we show *in vivo* that a single dose of CT1500 enhances re-entrainment to a shifted light dark cycle in a dose-dependent manner in mice and also phase shifts the circadian clock under constant dark with a clear time-of-day related pattern. The phase response curve shows CT1500 causes phase advances during the day and phase delays at dusk. Finally, we show that daily timed administration of CT1500 can entrain the circadian clock to a 24 h rhythm in free-running mice. Collectively, these data support the use of CT1500 in the treatment of disorders of circadian entrainment.

## Introduction

Circadian rhythms are endogenous oscillations in physiology and behaviour of approximately 24 h. Such rhythms allow an organism to anticipate daily changes in the external environment, and fine-tune biology to the varied demands of the astronomical day. In mammals, the core clock mechanism consists of a molecular transcriptional-translational feedback loop (TTFL) in which the transcription factors CLOCK and BMAL1 induce expression of their repressors *Per1/2* and *Cry1/2* (Partch et al., 2014). This core clock machinery appears to be present in every nucleated cell in the body. Peripheral clocks are aligned by the master circadian pacemaker in the suprachiasmatic nuclei (SCN) of the hypothalamus, resulting in a synchronised network of cell autonomous circadian oscillators driving rhythmic outputs. The SCN receives light input directly from the retina. The role of the eye, and in particular, that of photosensitive retinal ganglion cells (pRGCs) that express melanopsin (Foster et al., 2020) has been conclusively demonstrated by studies where the eye is removed (enucleation) (Foster et al., 1991) (Lee et al., 2003) or where pRGCs are ablated (Güler et al., 2008). These interventions result in animals free-running through the light/dark cycle, reminiscent of rhythms seen in individuals with eye loss and showing non-24 h sleep-wake rhythm disorder (N24SWRD) (Andrews et al., 2019). A thorough understanding of the mechanisms whereby light mediates entrainment will provide the substrate for the development of pharmacological interventions that mimic the effects of light on the circadian clock.

The mechanisms by which light acts on the circadian clock are broadly understood as a linear pathway from the eyes through to transcriptional changes in the SCN, recently reviewed in (Ashton et al., 2022). SCN cells receive light input *via* synaptic connections with the pRGCs. This then initiates a cell signalling cascade within SCN neurones which acts to reinforce, and if necessary adjust the phase of the clock, either by causing an advance or delay of the clock timing. Glutamate and PACAP, released from pRGC terminals act upon the molecular clockwork within SCN neurones *via* intermediary kinase cascades to converge on the cAMP response element binding protein (CREB), which is activated by phosphorylation at Ser133 and Ser142 (Ginty et al., 1993), resulting in the rapid and transient elevation of transcription of light-responsive genes such as *Fos, Dusp1 and Egr1,* whose expression is closely correlated with behavioural phase shifts (Jagannath et al., 2013a). The canonical clock genes *Per1* and *Per2* also exhibit a phase-dependent increase in mRNA expression, peaking around 1-2 h after light exposure, with *Per2* peaking later than *Per1.* The modulation of *Per1*/*2* by light is currently thought to be the pathway by which the core clockwork is regulated. However, the effects of light on the circadian clock are highly dynamic and dependent upon the time and intensity of light exposure, and other factors including sleep/wake history. How these different stimuli are integrated to achieve entrainment remains largely unknown.

For example, the time of light exposure will determine whether the phase of the clock is delayed or advanced, or whether the current timing is maintained, known as the phase response curve (PRC) (Pittendrigh and Daan, 1976). A few neurotransmitter and neuropeptide signalling pathways including melatonin (Redman, 1997), vasopressin (Yamaguchi et al., 2013) and adenosine (Antle et al., 2001; Jagannath et al., 2021) have been shown to modulate circadian entrainment (modifiers of entrainment reviewed in (Golombek and Rosenstein, 2010)). The important role of adenosine is considered in detail below.

The sleep/wake cycle is under the control of the circadian clock, but it is also driven by homeostatic sleep pressure, which accumulates during wakefulness and dissipates with sleep. As postulated by the two-process model, these two drivers were until recently believed to act largely independently, but recent work has led to the view that they do, in fact, interact. (Borbely, 1982; Borbély et al., 2016). Extracellular adenosine signals through four G-protein coupled receptors-A_1_ and A_3_ (Gi coupled) and A_2A_ and A_2B_ (Gs coupled). Extracellular adenosine in the brain builds up as a correlate of time awake and is thus a strong molecular correlate of the sleep homeostat (Porkka-Heiskanen et al., 1997), Adenosine signalling through A_1_ and A_2A_ receptors encodes sleep need (Lazarus et al., 2019). However, several groups have shown that adenosine, and adenosine receptor antagonists including caffeine, directly regulate circadian timing in several rodents and in humans, independently of their effects on sleep/wake physiology (Oike et al., 2011; van Diepen et al., 2014; Burke et al., 2015; Ruby et al., 2018). We previously described a signalling pathway downstream of adenosine receptors that directly regulates entrainment and identified adenosine A_1_/A_2A_ receptor antagonists that specifically altered clock gene expression and circadian rhythms *in vitro* and *in vivo via* the Ca^2+^-ERK-AP-1 pathway (Jagannath et al., 2021). Furthermore, we showed that the significance of adenosine signalling is to encode sleep/wake history to the clock and modulate its response to light in mice. In addition, the work presented in this paper, shows that adenosine receptor antagonists delivered to mice at specific times act like light to both phase-shift circadian rhythms, and also enhance re-entrainment to shifted light dark cycles (Jagannath et al., 2021). On the basis of these findings we suggest that this signalling pathway could provide a new therapeutic target for the stabilisation of circadian rhythm disorders, such as non-24 h sleep-wake rhythm disorder (N24SWRD). Here we characterise the clinically safe and orally bioavailable adenosine A_2A_/A_1_ receptor antagonist, CT1500 for its effectiveness as a chronomodulator in mice and show that CT1500, when administered as a single dose enhances re-entrainment to a shifted light dark cycle and phase shifts circadian rhythms in mice maintained under constant darkness (DD). Furthermore, when administered daily, CT1500 can effect stable entrainment under DD conditions.

## Materials and methods

### Animals

*Period2*::*Luciferase* transgenic mice were maintained in house and C57Bl6/J mice were sourced from Envigo. Only male mice were used for behavioural studies as females display scalloped circadian rhythms due to their oestrous cycle, which makes a true assessment of rhythmicity difficult in this sex. All studies were conducted on animals over 50 days of age. Animals were group housed with food and water *ad libitum* under a 12:12 h light:dark (LD) cycle, unless in an experiment where running wheel activity was recorded, in which case they were singly housed. All procedures were performed in accordance with the UK Home Office Animals (Scientific Procedures) Act 1986 and the University of Oxford’s Policy on the Use of Animals in Scientific Research (PPL 8092CED3). Animals were sacrificed *via* Schedule 1 methods in accordance with the UK Home Office Animals (Scientific Procedures) Act 1986 and approved by the University of Oxford Committee on Animal Care and Ethical Review (ACER).

### *In Vitro* tissue collection and Per2: Luc recording

Following euthanasia with isoflurane followed by cervical dislocation, the animal was enucleated and the brain was removed using standard methods. The brain was further blocked by removing the cerebellum and frontal cortex whilst preserving the integrity of the SCN and further sliced to 250 μm sections in NMDG aCSF using a compressotome (93 mm NMDG, 2.5 mm KCl, 1.2 mm NaH2PO4, 30 mm NaHCO3, 20 mm HEPES, 25 mm glucose, 2 mm thiourea, 5 mm Na-ascorbate, 3 mm Na-pyruvate, 0.5 mm CaCl2 and 10 mm MgSO4). The slices were further micro dissected under a microscope and transferred to membrane (Millicell Cell Culture Insert, 30mm, hydrophilic PTFE, 0.4 µm, Millipore) in recovery media consisting Hanks Balanced Salt Solution containing 100nm MK801, 2.5 mm AP-V and 3 mm Glutathione reduced ethyl ester for 1 h at 37° and then cultured in 500 µL DMEM containing B27 in a 5% CO_2_ incubator (methods described in Jagannath et al., 2021). Drugs at the indicated concentrations were added to the bath and the luciferase readout measured live. *Per2*: Luc rhythms were recorded from a BMG Labtech Fluostar Omega plate reader maintained at 36°C and readings taken from each well every hour. Data were then analysed using Multicycle rhythm analysis software.

### *In Vivo* drug administration

Intraperitoneal injection: The drugs were formulated in a vehicle consisting of 5% Koliphor-HS15 (Sigma-Aldrich, UK) 5% Cyclodextran (Sigma-Aldrich, UK) in 0.9% saline. This vehicle was warmed to 37°C and the indicated drugs kept at a 100x stock in DMSO and mixed at the appropriate concentration to be administered at approximately 10 ml/kg intraperitoneal injection.

*Oral gavage*: The drugs were formulated as a suspension in 10% sucrose (w/v) and 0.3% Tween 80 (v/v) in water. A weighed quantity of drug was added to an Eppendorf tube and wetted with a small quantity of vehicle and initially made into a smooth paste using a pestle. The paste was then made up to final quantity with vehicle and homogenised in an ultrasonic bath in short bursts for up to 32 min until visibly homogeneous. Formulations were dispensed into amber glass bottles for dosing and stored refrigerated (2°C–8°C). Formulations were stirred from at least 15 min before the start of dosing until the completion of their use for dosing, to ensure thorough re-suspension and homogeneity.

When the time point of administration was in the dark, the procedure was conducted under dim red light.

### Behavioural assays

In mice, the activity of the circadian clock is most commonly determined by measuring behavioural outputs, such as levels of locomotor activity. Voluntary wheel running activity can be measured non-invasively over long periods of time, providing information on the state of the core circadian clock, entrainment and phase shifting (Albrecht and Foster, 2002) (Jud et al., 2005). Animals were housed in commercially available light-tight chambers and activity recording equipment from Actimetrics (Wilmette, IL) used.

#### Re-entrainment assay

C57Bl6/J male mice (*n* = 10–20, 80 days or older) were maintained as singly housed in shoe-box cages equipped with running wheels in light tight chambers on a 12:12 L:D cycle (100 lux from white LED lamps). On stable entrainment (typically achieved within 1 week under a stable light dark cycle), at ZT6 (Zeitgeber Time 6, which is six hours after the onset of light), the animals received an intraperitoneal injection (10 ml/kg) of drug constituted as above. The L:D cycle was immediately advanced by 6 h, simulating landing in a time zone 6 h ahead. Onset of activity on each day was used to measure phase relative to the L:D cycle, data analysed on Clocklab (Actimetrics, Wilmette, IL), and animals were allowed up to 2 weeks to entrain to the new LD cycle. On achieving stable entrainment, the animals underwent the same procedure with the specific dose/treatment rotated between the subjects. Up to 4 rotations were undertaken on any one animal, after which the animal was sacrificed. N = 10–20 for each condition.

#### Phase shift assay

C57Bl6/J male mice (*n* = 60, 80 days or older) were first maintained on running wheels in light tight chambers on a 12:12 L:D cycle. On stable entrainment, the animals were released into constant dark (12:12 D:D) and allowed to free-run. Each animal was dosed with the indicated drugs at 10 am once weekly for up to 9 weeks. At the start of the protocol, 10 am corresponded with ZT4 (lights on at 6 am), but as the mice were free-running, we expected 10 am would correspond with a range of ZTs over the following weeks, such that over the course of the experiment, the animals would receive drugs/light across the 24 h timescale, in order to construct an approximation of the phase response curve (PRC). In half the cases, drug administration was combined with a 10 lux 30-minute light pulse, in order to assess the combined effects of CT1500 and light at different times. This intensity was chosen, as it is dim enough to be not saturating for phase shifting responses (Freedman et al., 1999) and that any enhancement of the effects of light by CT1500 would be detected. The combination of treatments (drug, drug + light, vehicle, vehicle + light) were rotated between groups of ten animals, such that no animal received the same treatment any consecutive weeks and such that all combinations were tested in all animals, albeit at different time points. Onset of activity was used to plot phase using the 6 days on either side of the drug administration and the difference in phase calculated.

#### Entrainment assay

C57Bl6/J male mice (*n* = 5/6 per group, 80 days or older) were first maintained on running wheels in light-tight chambers on a 12:12 L:D cycle. On stable entrainment, the animals were released into constant dark (12:12 D:D) and allowed to free run. This resembles the condition seen in individuals with non−24 h sleep wake disorder (Salva et al., 2017), where the clock is not entrained. The mice were then dosed daily at 10 am, which coincided with CT8 at the start of the protocol (lights on at 6 am when the mice were housed under L:D), with the indicated drugs for up to 15 days, after which the animals were allowed to free run. Period length was estimated at the indicated times through the experiment.

### Statistical analysis

Data presented as individual replicates or mean ± SEM, and n represents the individual replicates per group, as detailed in each legend. GraphPad Prism 9 was used to conduct all statistical analysis, with the details of the tests and their results included in each legend.

## Results

In this study, we profiled the effects of several A_2A_/A_1_ antagonists with greater A_2A_ activity, but also some activity at A_1_ for their ability to alter circadian rhythms in mice. This profile of drug was chosen, as our previous work (Jagannath et al., 2021) showed that antagonists that acted at both receptors were more effective at phase shifting the clock than antagonists that targeted either receptor alone. Several of these molecules have been developed for their application in Parkison’s disease, where A_2A_ receptors are co-expressed with and counteract the function of Dopamine D2 receptors (Shook and Jackson, 2011). Of these, a preclinical tool molecule that we previously studied for its effect on the clock JNJ-40255293 (Jagannath et al., 2021) (Atack et al., 2014) and three further compounds that have been shown to be clinically safe (Pinna, 2014), namely CT1500 (ST1535) (Stasi et al., 2006; Stasi et al., 2015), CT1500s active metabolite CT1517 and SCH 420814 (Neustadt et al., 2007), were used in this study (Table 1).

**TABLE 1:** The compounds used in this study, their structure and activity at the different adenosine receptors are detailed.

Compound (CAS ID)	Structure	EC_50_ nM	EC_50_ nM	EC_50_ nM	EC_50_ nM
		A_2A_	A_1_	A_2B_	A_3_
JNJ-40255293 (Atack et al., 2014) (147,271-25-7)	, 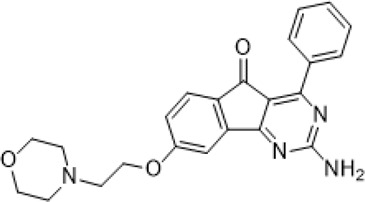	48 ± 16	6.5 ± 3.8	230 ± 92	9,200
SCH 420814 (Neustadt et al., 2007) (377,727-87-2)	, 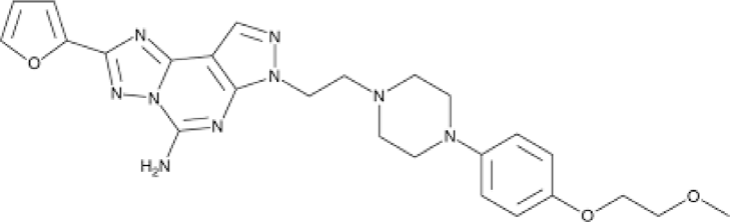	1.1–2.5	>1,000	>1,700	>1,000
CT1500 (Stasi et al., 2006, 2015) (496,955-42-1)	, 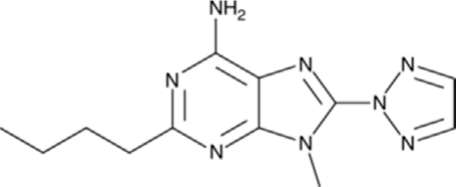	8 (5–14)	103 (59–181)	353	>1,000
CT1517 (CT1500 metabolite) (1,246,018-36-9)	, 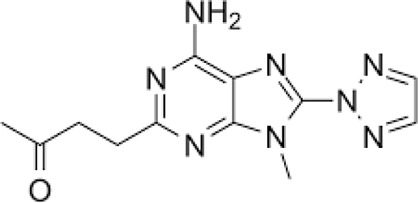	12 (7–23)	197 (131–206)	–	–

**FIGURE 1:**
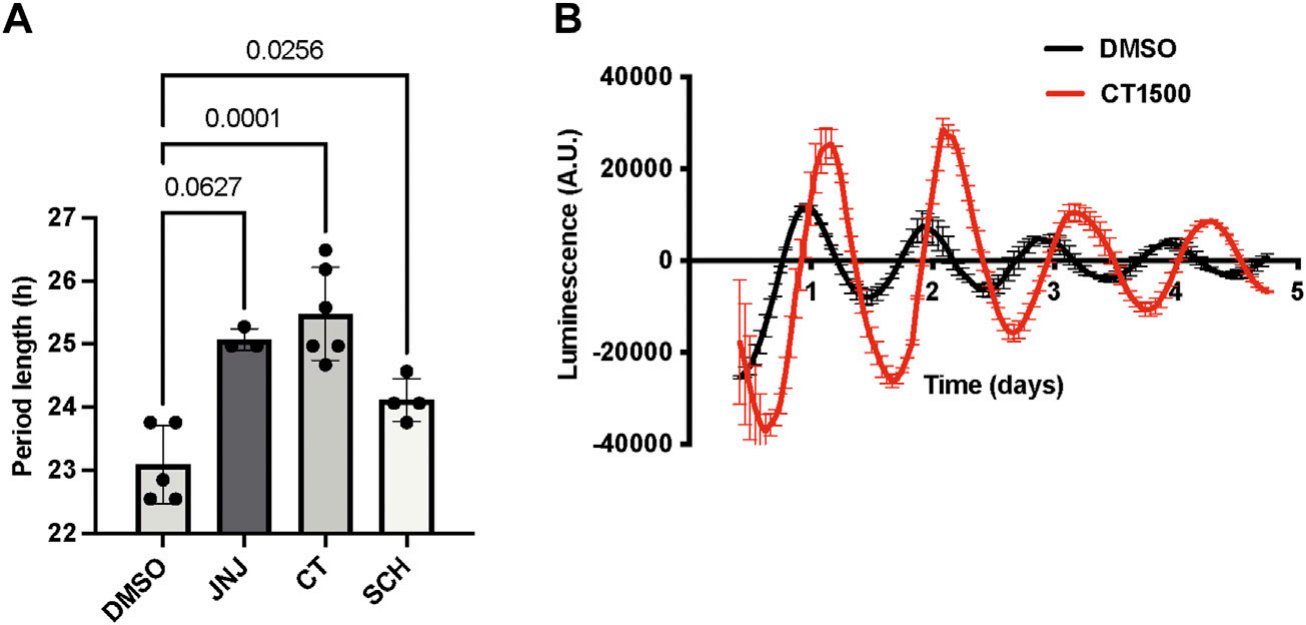
Effect of CT1500 on period length of SCN. **(A)** SCN isolated from adult *Per2::Luc* mice were cultured for 3 days after which the indicated drugs were added to the bath at a final concentration of 3 µM (n as indicated in figure) and bioluminescence recorded for another 5 days. Period length after drug addition reported and data analysed by one-way ANOVA (*p* = 0.001, n too small to determine normality). *p*-values from multiple comparisons with Tukey’s multiple comparisons test reported in the figure. **(B)** Representative trace from a slice treated with CT1500 shown. Data presented as each replicate and mean and S.D. overlaid.

We validated the effects of the specific adenosine receptor antagonists as listed in Table 1 on the SCN *ex vivo* slice cultures from *Per2::Luc* transgenic mice (Yoo et al., 2005), which express a *Per2::Luciferase* reporter gene. A_2A_/A_1_ antagonists were added to the bath and the luciferase readout measured live. CT1500 lengthened period (Figure 1A) by 1.17 ± 0.37 h (mean ± SEM), compared to pre-drug. Similar period lengthening was also observed with JNJ4025529 (JNJ)3 and SCH 420814 (Figure 1B), although escaping statistical significance with JNJ in this particular experiment.

**FIGURE 2:**
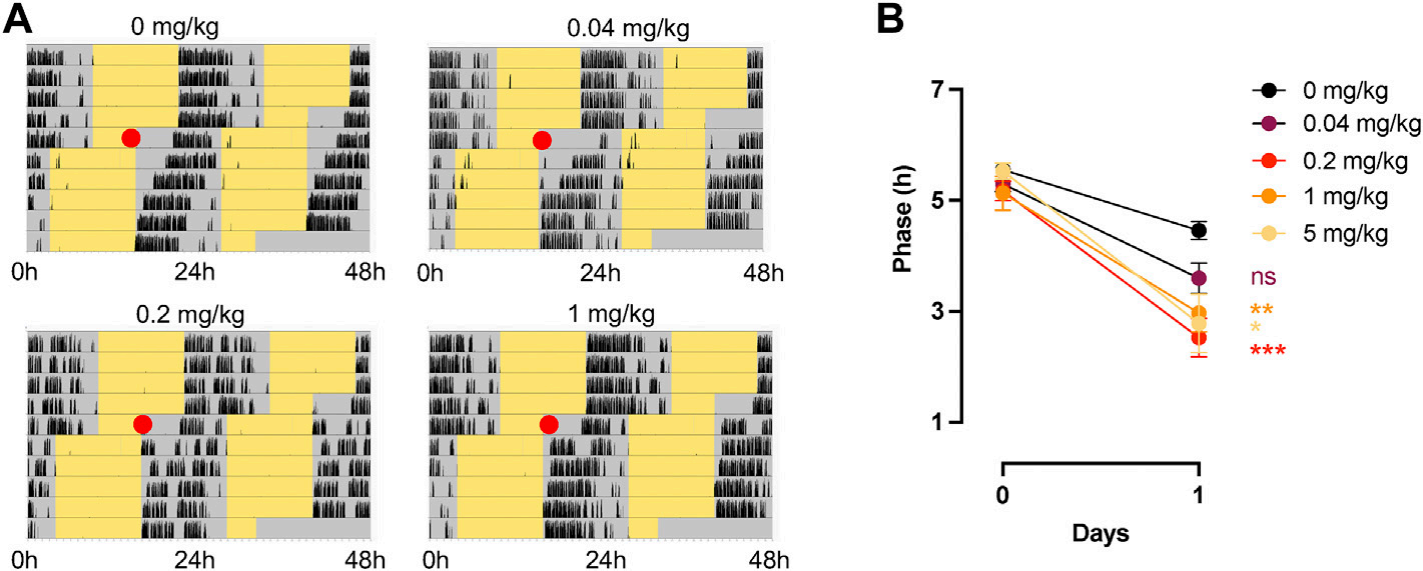
Effect of CT1500 on photic re-entrainment. **(A)** C57Bl6/J (*n* = 10–12 per group) male mice housed in a 12:12 LD (Light Dark) cycle, yellow indicates lights on, grey lights off, and black wheel running activity and at the red dot (ZT6) received a single indicated dose of CT1500 and the LD cycle immediately advanced by 6 h. Representative actograms shown. **(B)** Phase of activity onset relative to lights off on the subsequent day plotted and data analysed (data were found to follow a normal distribution, Anderson-Darling test) by Two-way ANOVA. A significant effect of dose was observed (F = 242, *p* < 0.001), Tukey’s multiple comparisons tests are as follows (0.04 mg/kg *vs*. 0—difference = 0.8616, adj. *P* = 0.43; 0.2 mg/kg *vs*. 0—difference = 1.929; adj. P = 0.0001; 1 mg/kg *vs*. 0—difference = 1.486, adj. P = 0.004; 5 mg/kg vs. 0—difference = 1.670, adj. P = 0.024. The different doses did not statistically separate from one another, however a trend towards higher efficacy at mid-range was observed, indicating a bell-shaped dose response curve. Data presented as mean + - S.E.M.

We next tested CT1500, CT1517 and SCH 420814 for their ability to enhance re-entrainment to a shifted L:D cycle, as previously shown with JNJ42055293 (Jagannath et al., 2021). Several “non-photic” cues that rely on cAMP or MAPK/ERK signalling (which are also part of the photic signalling cascade) including melatonin and exercise cause phase advances of the clock *in vitro* and *in vivo* when administered during the subjective day (Prosser and Gillette, 1989; Reebs and Mrosovsky, 1989; Biello and Mrosovsky, 1996; Lewy et al., 1998). Importantly, some of these have also been shown to enhance photic resetting presumably by impinging at some level on the same signalling pathways as light (Agostino et al., 2007; Pilorz et al., 2014), as we showed previously with JNJ40255293 (Jagannath et al., 2021). We first conducted a pilot study with *n* = 3 mice per group, testing CT1500 at a range of doses guided by previous work which dosed this compound at 10 mg/kg (Stasi et al., 2006, 2015)—0.2 mg/kg, 5 mg/kg and 25 mg/kg. Surprisingly, this experiment indicated that of these doses, 0.2 mg/kg had the greatest effect on enhancing re-entrainment to a shifted light:dark cycle (data not shown), with the difference in phase relative to lights off on the day after (day 1) drug administration (day 0 being the day of administration) being the readout. Our further studies using larger numbers of mice (*n* = 10–20) therefore used 0.04, 0.2, 1 and 5 mg/kg doses (Figure 2A), where we noted that whilst 0.04 mg/kg dose was not effective over vehicle administration, 0.2 mg/kg and 1 mg/kg were the most effective, with a trend towards reduced efficacy at 5 mg/kg (Figure 2B). This demonstrated a bell-shaped dose response curve. CT1517 (Supplementary Figure S1A) and SCH 420814 (Supplementary Figure S1B), also enhanced photic re-entrainment, where the latter showed a slightly higher dose requirement, with 1 mg/kg showing the highest efficacy. This is perhaps in line with the different profiles of these compounds, SCH 420814 has less activity at A_1_ when compared with CT1500 or CT1517 (see Table 1).

**FIGURE 3:**
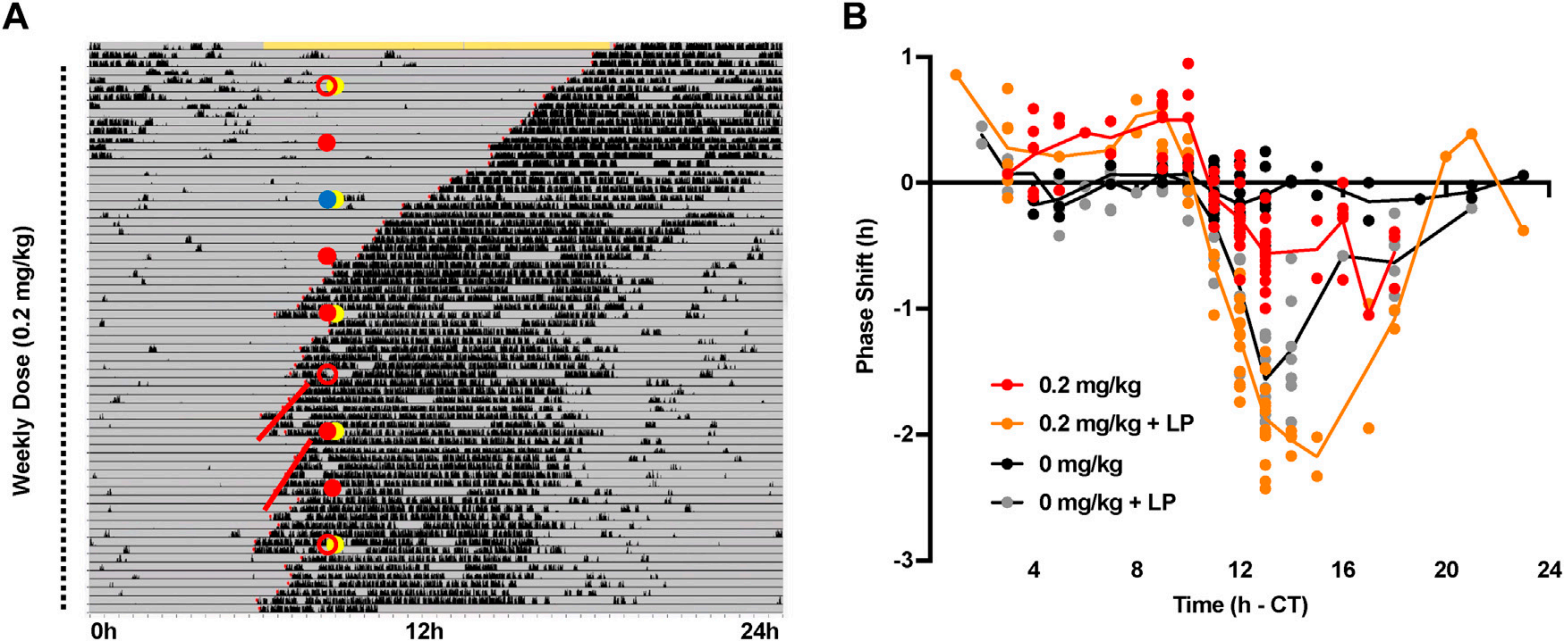
Phase response curve to CT1500 with and without a simultaneous light pulse. **(A)** C57Bl6/J (*n* = 40) male adult mice were housed in constant dark, representative actogram shown. Grey indicates darkness, black bars are wheel running activity. At each dot (10 am), the mice received an 0.2 mg/kg CT1500 (red filled) or vehicle (0 mg/kg red open), in combination with a 10 lux 30-minute light pulse (yellow filled) or no light pulse. Blue circle indicates a cage change co-inciding with treatment, data not included for this week. The treatments were rotated between animals weekly for nine weeks. The onset of activity on the 6 days prior to and subsequent to injection (red lines) was used to determine phase shift caused by CT1500 alone, or in combination with light **(B)** Phase shifts from each individual test plotted relative to the time of administration. A two-way ANOVA shows a significant effect of treatment on the phase shift. As data were not normally distributed, multiple t-tests (Mann-Whitney, non-parametric) with two-stage step up FDR correction were used to analyse data. CT1500 caused significant advances (*p* < 0.05) at CT8-9 and CT14. Data presented as each replicate with a line connecting the mean.

We then conducted a partial phase response curve (PRC) to timed administration of CT1500. CT1500 was administered at the lowest effective dose (0.2 mg/kg intraperitoneal) at a range of time points across 24 h in free running mice housed in DD (*n* = 60), (Figure 3A). CT1500 or vehicle was administered to the animals with or without the simultaneous application of a 10 lux 30-minute light pulse, to assess the interaction between adenosine receptor antagonism and light on circadian entrainment. The combinations of treatments were rotated between the animals such that no animal received the same treatment 2 weeks consecutively. Not all time points could be covered by this protocol, with very few treatments administered in the CT21 - CT2 window. Thus the PRC constructed is partial, nevertheless it provided valuable information on the relation between CT1500 efficacy and time of administration. We found that CT1500 caused an approximately 30-minute phase advance when administered CT8-11, and a 30-minute phase delay at CT14-16. Light as expected resulted in a 1–1.5 h phase delay at the same window (CT13-17). Interestingly, CT1500 enhanced the effects of light in the same window (CT13-17), resulting in 2–2.5 h delay, but this effect was not observed at CT8-11. An area under curve analysis confirmed that CT1500 had a largely similar effect size to a dim light pulse, and that CT1500 also greatly enhanced the phase delaying effect of light (Supplementary Figure S2). In summary, these results suggest CT1500 has distinct effects on circadian phase depending on time of administration.

**FIGURE 4:**
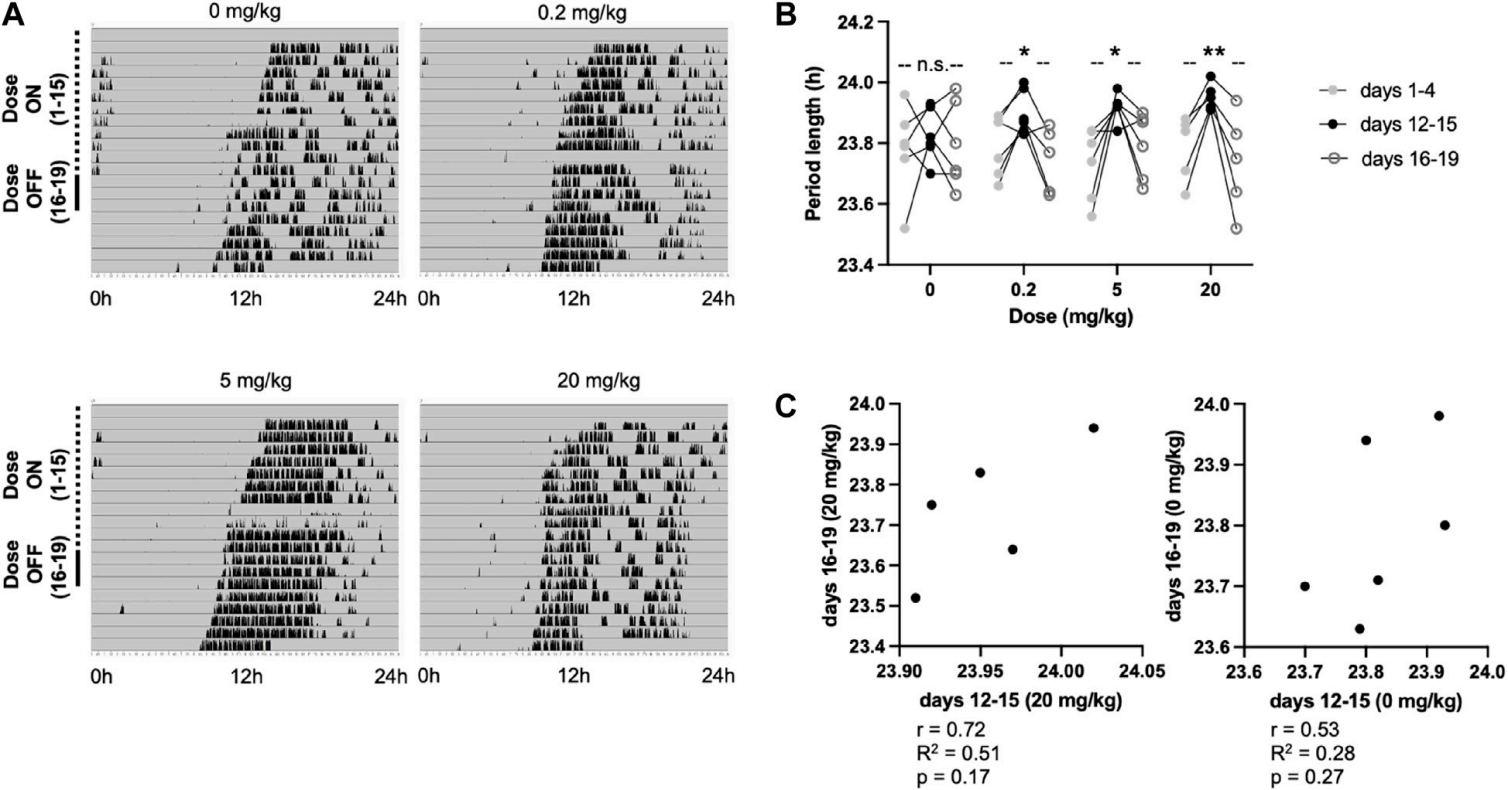
Effect of CT1500 on entrainment in free-running mice. **(A)** C57Bl6/J (*n* = 5/6 per group) male mice housed constant dark (grey) and wheel running activity indicated in black. The animals received a single oral dose of the indicated treatment at 10 am every day marked in the dotted line, and no treatment during the days marked with the solid line. Represented actograms shown **(B)** Period length across the indicated days analysed with One way ANOVA which indicated a significant effect of drug (n too small to calculate normality). Two-stage linear step-up procedure of Benjamini, Krieger and Yekutieli multiple comparisons * (*p* < 0.05) or ** (*p* < 0.01) indicate a significant increase in period on days 12–15 (on treatment) relative to days 16–19 (off treatment) **(C)** Correlation of period length on drug (days 12–15) versus after withdrawal (days 16–19) indicated for two doses, showing no significant correlation (Pearson’s r, R2 and *p*-value indicated).

Given the mechanism of action of A_2A_/A_1_ antagonism on the circadian clock, we hypothesised that daily administration of CT1500 could provide an entrainment cue in constant dark. As a pilot experiment showed that mice entrained to daily intraperitoneal injections of vehicle very often, we needed an alternative, less severe method of administration. We assessed the efficacy of oral dosing by gavage in the re-entrainment assay. We found broadly similar results as with intraperitoneal injection (Supplementary Figure S3), and additional experiments confirmed that animals did not entrain to daily oral gavage. As a result, this route was used to perform the entrainment assay. 0.2, 5 and 20 mg/kg doses were administered once daily at 10 am, which coincided with CT8 at the start of the experiment, for 15 days (Figure 4A), CT8 being among the best times of administration for causing a phase advance as seen in Figure 3. Period length was estimated at the start of dosing, end of dosing and for the 3 days immediate after dosing had ended. Whilst all three doses increased period length to close to 24 h and resulted in clear entrainment patterns, the highest dose, 20 mg/kg resulted in an approximately 24 h rhythm consistently (Figure 4B). Furthermore, there appears to be a correlation between period on drug and after withdrawal such that an increased period length on drug results in an increased period length that persists after withdrawal, although this was not statistically significant in our analysis, presumably as the power of the experiment was not sufficient (Figure 4C).

## Discussion

In this study, we show that the adenosine A_2A_/A_1_ antagonist CT1500, and related compounds CT1517 and SCH 420814 regulate circadian entrainment by enhancing re-entrainment to a shifted light dark cycle, phase shifting the clock in time-dependent manner and also mediating entrainment when administered daily. CT1500 is a clinically safe, well tolerated compound that is orally bioavailable and brain permeant, making it an ideal candidate for clinical progression. Collectively, the data presented support the use of CT1500 in the treatment of disorders of circadian entrainment such as N24SWRD in the blind (Salva et al., 2017), and across the neurodegenerative, neurodevelopmental and psychiatric disorder spectrum, where sleep and circadian rhythm disruption is prevalent (Jagannath et al., 2013b). CT1500 would also be beneficial to enhance the effect of correctly timed light exposure in sighted individuals such as in jet lag disorder or shift work disorder (Reid and Abbott, 2015).

Adenosine antagonists are not the only clinical compounds that can be used as chronomodulators-Melatonin receptor agonists currently remain the only approved treatment for circadian entrainment disorders, specifically, N24SWRD (Lockley et al., 2015). However, limited efficacy in a broad spectrum of clinical populations has stressed the need for alternative treatments [reviewed in (Zee, 2010) (Foster, 2021)]. Multiple compounds targeting either core clock components, such as activators of Cryptochrome (Hirota et al., 2012) or the ancillary loop-RORs and Rev-Erbs—[reviewed in (Sulli et al., 2018)] have been developed. The majority of these suffer from poor bioavailability and blood brain barrier (BBB) permeability. Multiple drugs that modulate neurotransmitter signalling, including serotonin, GABA, acetylcholine and dopamine also modify phase-shifting in response to light, and as these pathways have been targeted primarily for CNS indications, BBB permeability is less of an issue. Lee et al. have recently reviewed over 150 studies in this area (Lee et al., 2022) and found that the vast majority of such CNS relevant compounds suppress light response, or only weakly enhance them. The exceptions include 5HT1A agonists/mixed agonist-antagonists (Lee et al., 2022) and the cGMP phosphodiesterase inhibitor siledafil (Agostino et al., 2007); both of which have undesirable peripheral and central side effects. Specific adenosine A_2A_/A_1_ antagonists however have few contraindicated off-target effects.

CT1500 would be expected to increase arousal and locomotor activity in line with A_2A_ antagonism (Huang et al., 2005; Rose et al., 2006), although at the doses used, we did not see any significant changes in wheel running activity. Furthermore, A_2A_ receptors functionally interact with mGlu5 and Dopamine D2 receptors, and this would also have effects on behaviour (Pinna, 2014). These effects should be considered together with the circadian efficacy when progressing to clinical studies, particularly in deciding the optimum time of administration. Indeed, increased arousal/activity during the subjective day may be a beneficial side effect, and has a reduction of daytime sleepiness, with no negative impact on night-time sleep has been reported when another A_2A_ antagonist, istradefylline, was administered in the morning to patients with Parkinson’s disease (Suzuki et al., 2017).

These findings raise the question as to why caffeine cannot be used to effect entrainment. Caffeine has drawbacks including multiple off-target effects (including inhibition of phosphodiesterase resulting in peripheral off target effects), a long half-life, and long-term administration results in desensitisation at adenosine receptors. Furthermore, a previous study reported that daily caffeine dosing in three blind individuals with N24SWRD did not entrain their rhythms, but did improve daytime alertness (st. Hilaire and Lockley, 2015), which may at first pass appear contraindicatory, especially in light of the robust evidence that caffeine does alter circadian rhythms in mice (Oike et al., 2011; van Diepen et al., 2014; Ruby et al., 2018). However, long term caffeine administration causes tolerance, secondary to its antagonistic activity at the adenosine receptors (Fredholm et al., 1999; Svenningsson et al., 1999). This rapid desensitisation is avoided with synthetic adenosine receptor antagonists (Halldner et al., 2000). Given the participants in the trial were habitual coffee drinkers (6–15 cups) it is possible that tolerance was an issue. Furthermore, given the long half-life of approximately 5 hours, it is conceivable that caffeine acts across a broad section of the phase response curve, thus resulting in blunted shift (an advance during the day, followed by a delay at dusk). The above, alongside many other reasons, may also explain the bell-shaped dose response curve we observe in the re-entrainment assay. For example, from our previous work, we noted that compounds that had a high efficacy at A_2A_ but also some efficacy at A_1_ were the most effective at increasing clock gene expression and enhancing re-entrainment (Jagannath et al., 2021). Higher doses may alter receptor occupancy in a manner that is no longer optimal in this particular assay. However, it is important to note that a higher dose range does result in increased efficacy in the entrainment assay in constant dark, where there is no effect of light.

## Data Availability

The raw data supporting the conclusion of this article will be made available by the authors, without undue reservation.
