# Supplementary figures and images for: The regulation of circadian entrainment in mice by the adenosine the A 2A /A 1 receptor antagonist CT1500
Source: Front Physiol. 2022 Dec 20;13:1085217. doi: 10.3389/fphys.2022.1085217 (PMC9808084; doi:10.3389/fphys.2022.1085217)

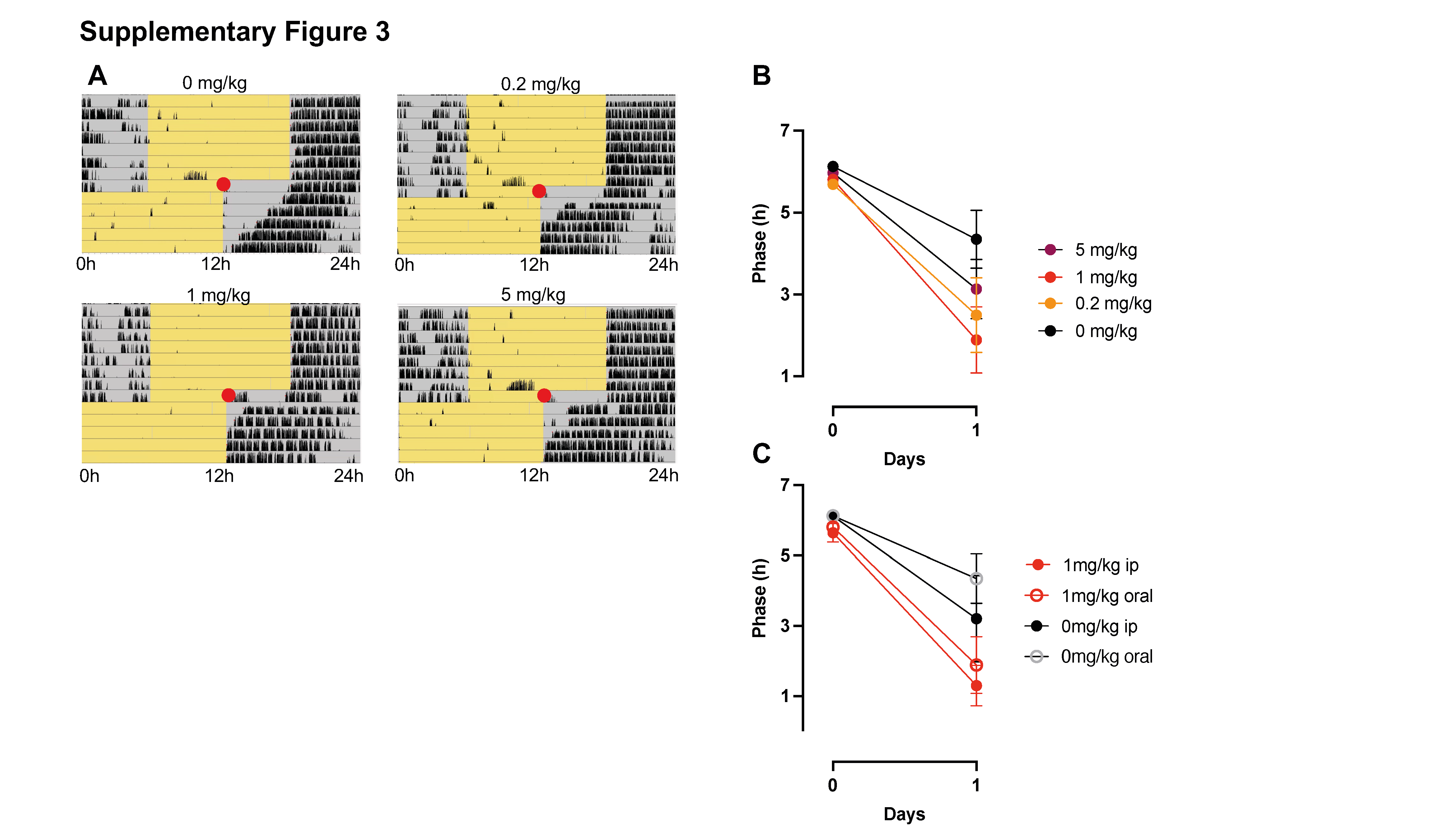

Supplement: Supplementary file 1 [file Image3.TIFF]

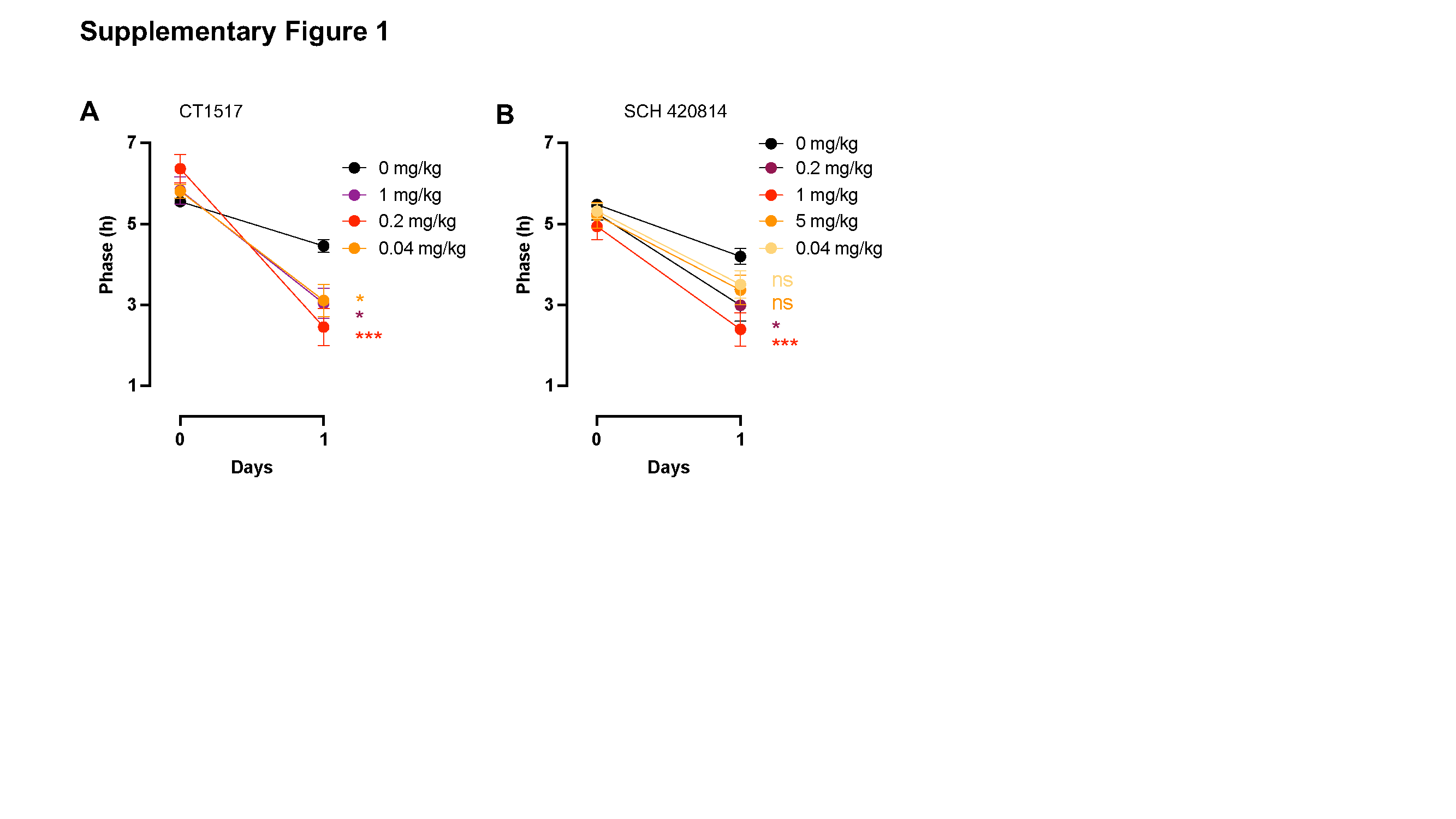

Supplement: Supplementary file 2 [file Image1.TIFF]

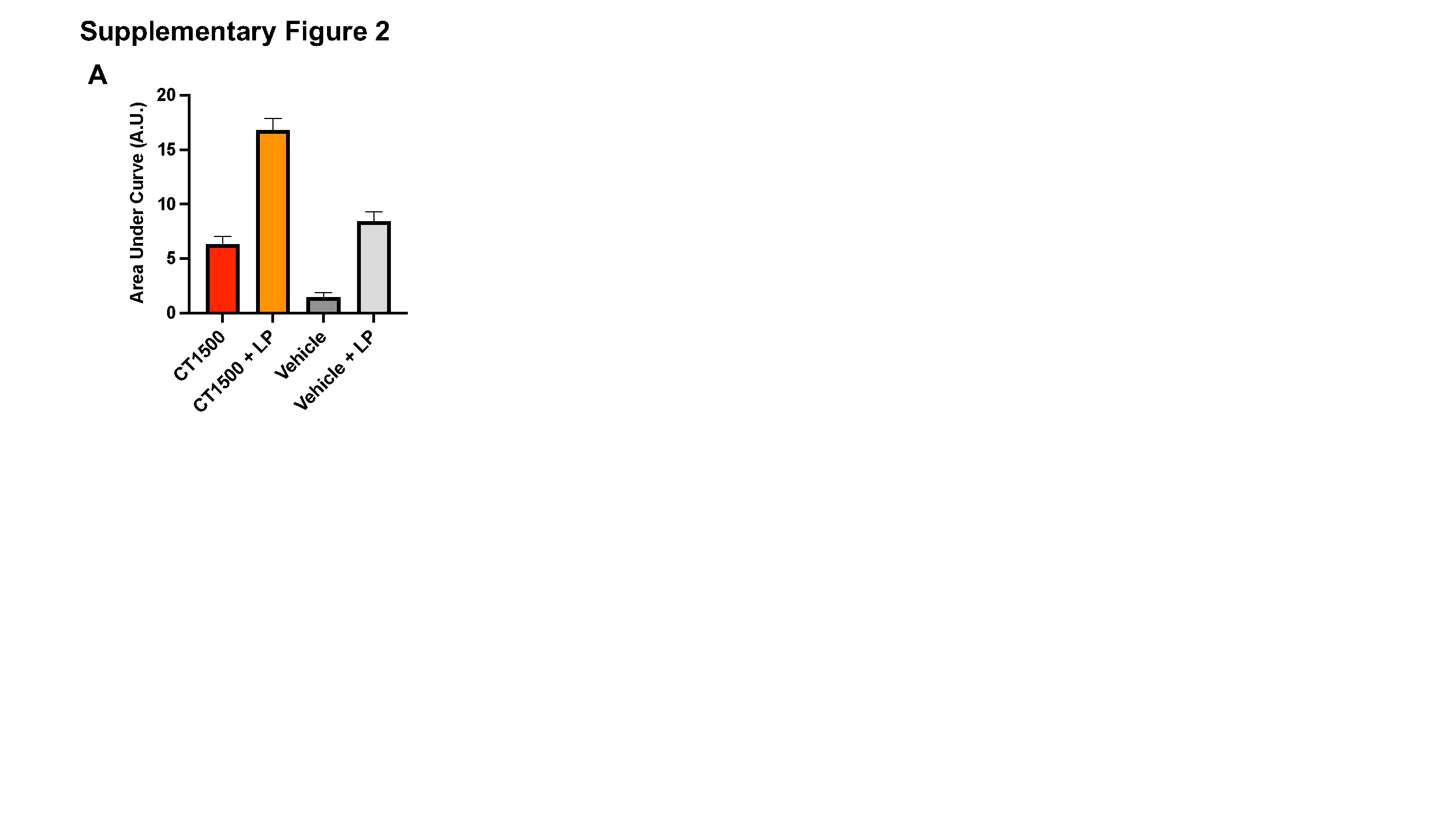

Supplement: Supplementary file 3 [file Image2.TIFF]
